# Therapeutic Plasma Exchange for Treatment-Resistant Tumefactive Demyelinating Lesion: A Case Report

**DOI:** 10.7759/cureus.53786

**Published:** 2024-02-07

**Authors:** Salem Vilayet, Eily Hayes, Munsef Barakat, Milos Budisavljevic, Anand Achanti

**Affiliations:** 1 Division of Nephrology, Medical University of South Carolina, Charleston, USA

**Keywords:** steroid-resistant multiple sclerosis, autoimmune neurological disorders, clinically isolated syndrome, therapeutic plasma exchange (tpe), tumefactive demyelinating lesions

## Abstract

Tumefactive demyelinating lesions (TDLs) can present as an isolated clinical incidence or could represent the initial presentation of multiple sclerosis. Radiological TDLs are characterized by large tumors like >2 cm space-occupying lesions with mass effect and perilesional edema. Diagnosis is based on MRI imaging and extensive work to exclude other causes and a biopsy of the lesion is often required. First-line treatments include pulsed methylprednisolone. We present a case of a refractory TDL treated successfully with therapeutic plasma exchange.

## Introduction

Multiple sclerosis (MS) is one of the most common neurological autoimmune diseases. It commonly affects females more than males and can have a variable clinical course, including clinically isolated syndrome, relapsing-remitting, primary progressive, and secondary progressive. Clinically isolated syndrome represents the first episode of MS and demyelinating lesions in the absence of clinical or radiological evidence of prior lesions [1].

Although the etiology of MS is unknown, it's widely accepted that it is caused by an immune dysregulated response in the presence of genetic predisposition and an environmental trigger such as Epstein-Barr virus (EBV) infection, vitamin D deficiency, and smoking [2]. Clinically, MS presents with variable neurological signs and symptoms depending on the location of the demyelinating lesion. Symptoms include painful visual impairment due to optic neuritis, hemiparesis, and hemiplegia, along with sensory symptoms and bladder dysfunction.

Tumefactive demyelinating lesions (TDL) are a rare radiological entity and considered to be a variant of MS that is characteristically aggressive and presents with large (>2 cm) tumor-like demyelinating lesions seen on magnetic resonance imaging (MRI) [3]. TDLs can present in patients with previously established MS or represent a clinically isolated syndrome [3]. Given the size and inflammatory nature of the lesion, it can cause significant perilesional edema and a mass effect and could manifest as relapsing and remitting MS resistant to standard therapies.

We present a case of newly diagnosed tumefactive MS that was initially treated with IV methylprednisolone without benefit and subsequently treated successfully with therapeutic plasma exchange (TPE).

Part of this article and the case presentation were previously presented at the American Society for Apheresis 44th Annual Meeting, April 26-28, 2023 [4].

## Case presentation

A 30-year-old female with no significant past medical history presented with 3 weeks of subjective left upper and lower limb weakness and paresthesia along with pins and needle sensation on the same side, her symptoms worsened over the period to the extent that she felt that it was affecting her daily function. She didn’t report any back pain, visual symptoms, or urinary or bowel dysfunction, with no history of fever or constitutional symptoms and no recent viral illness or immunization.

On examination, the patient was clinically well, afebrile, and hemodynamically stable. A neurological examination showed normal and intact higher brain function with intact cranial nerve examinations, no appreciated visual field defect, and normal visual acuity. Further examination revealed intact deep tendon reflexes 2+ with mild left-side upper and lower limb weakness more prominent in the left leg with strength 4/5 and intact sensation. The right side showed normal tone, power, and reflexes. Babinski was negative bilaterally with a normal gait and cerebellar examination. Initial CT head without contrast showed a parietal space-occupying lesion with evidence of vasogenic edema.

**Figure 1:**
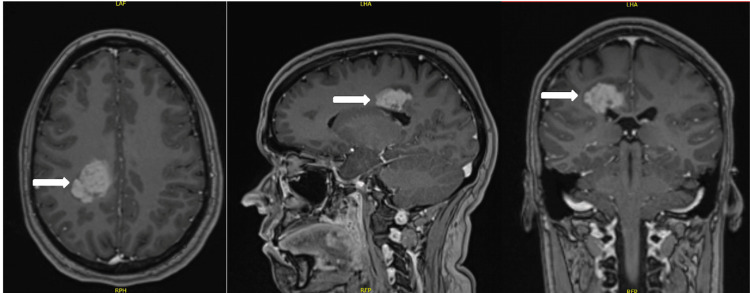
White arrow indicating enhancing periventricular white matter lesion centered within the right posterior cingulate

She was subsequently started on steroids, initially for the concern of vasogenic edema, and underwent an MRI of the brain for further evaluation. The MRI showed a 3.3×2.4×2.3 cm-enhancing periventricular white matter lesion centered within the right posterior cingulate (Figure 1).

MRI spectroscopy showed that the lesion has a diffusely increased choline peak with an N-acetyl aspartate (NAA): creatine ratio of 3.48 and choline: creatine peak of 4.2 without lactate peak and no myoinositol peak detected. MRI spine was done and reported as normal.

Findings from extensive blood work performed to rule out other causes were all negative and included QuantiFERON, hepatitis panel, anti-aquaporin-4 antibody (AQP4), myelin oligodendrocyte glycoprotein (MOG), angiotensin-converting enzyme, Epstein-Barr virus, cytomegalovirus, *Borrelia burgdorferi* antibody, and Venereal Disease Research Laboratory (VDRL). CSF analysis was notable for glucose of 77 mg/dL, protein of 21.8 mg/dL, negative xanthochromia, an absolute WBC of 5, CSF oligoclonal bands were 9 bands (reference range: <2 bands), and cytology for malignant cells was negative (Tables 1-2 show the workup during the admission).

**Table 1:** Laboratory values obtained during the admission WBC: white blood cell count; BUN: blood urea nitrogen; ALT: alanine transaminase; AST: aspartate aminotransferase; NMO/AQP 4: anti-aquaporin-4 antibody

Blood Test	Value	Reference Range
Hemoglobin	12.8 g/dL	13-15 g/dL
WBC	10 k/cumm	4-11 k/cumm
Platelets	179 k/cumm	150-400 k/cumm
Serum creatinine	0.7 mg/dL	0.7-1.2 mg/dL
BUN	12 mg/dL	8-26 mg/dL
Serum sodium	139 mmol/L	135-145 mmol/L
Serum potassium	4 mmol/L	3.5-5.1 mmol/L
Serum calcium	9.6 mg/dL	8.4-10.3 mg/dL
Serum bicarbonate	25 mmol/L	22-29 mmol/L
Serum chloride	105 mmol/L	98-108 mmol/L
Magnesium	2.1 mg/dL	1.6-2.6 mg/dL
Phosphorus	3.5 mg/dL	2.3-4.7 mg/dL
ALT	20 U/L	<34 U/L
AST	16 U/L	<34 U/L
Alkaline phosphatase	53 U/L	35-150 U/L
Total bilirubin	0.7 mg/dL	0.2-1.2 mg/dL
Serum myelin oligodendrocyte glycoprotein (MOG) antibodies	Negative	Negative
Serum NMO/AQP4 antibodies	Negative	Negative

**Table 2:** Lumbar puncture result and CSF analysis NMO/AQP4-IgG: anti-aquaporin-4 antibody; VDRL: Venereal Disease Research Laboratory test; CMV PCR: cytomegalovirus polymerase chain reaction

CSF Analysis	Value	Reference Range
Color	Colorless	Colorless
Clarity	Clear	Clear
Protein	21.8 mg/dL	15-45 mg/dL
Glucose	77 mg/dL	40-70 mg/dL
Absolute WBC	5/cumm	0-5/cumm
RBC	0/cumm	non
Xanthochromia	Absent	Absent
oligoclonal band	9 Bands	<2 Bands
NMO/AQP4-IgG	Negative	Negative
Borrelia burgdorferi antibodies	1019	1003-1030
VDRL	Negative	
CMV PCR	Negative	
Angiotensin Convert enzyme	Negative	

She underwent a brain biopsy, result was notable for non-specific abundant macrophages along with reactive gliosis, the subsequent tissue examination was negative for the JC virus and *Toxoplasma gondii*. A diagnosis of TDL was made and started on pulse methylprednisolone 1g IV for 5 days, followed by oral taper.

During the hospital stay, the patient continued to have worsening left-sided paresthesia, heaviness, and worsening headaches. An MRI of the brain was repeated, which didn’t reveal any worsening of the lesion or the surrounding edema. Given the lack of significant improvement on the pulse steroid, she was subsequently started on TPE using the Spectra Optia continuous-flow centrifugal apheresis system, using 5% albumin replacement every other day for five sessions, with complete resolution of her paresthesia after the 3rd TPE session and improvement of her power. On a 3-month follow-up after the initial presentation, the patient reported total resolution of her initial symptoms and planned to continue clinical and radiological observation.

## Discussion

MS is a chronic, inflammatory disease that causes demyelinating lesions, with a usual course of relapses followed by remissions at onset. Disability may accumulate rapidly in cases of severe relapses with incomplete recovery, but any relapse can result in disability accumulation and deterioration of neurological functional status. Clinically, tumefactive MS presents with similar signs and symptoms to typical MS with smaller demyelinating plaques, but transcranial magnetic stimulation (TMS) symptoms tend to be more severe with more dense neurological deficits due to the size of the lesions and significant perilesional edema. Other symptoms that can occur are elevated intracranial pressure, seizures, and cortical symptoms due to cortical involvement.

The diagnosis of TMS can be challenging and the differential can be long; this includes other demyelinating diseases, acute disseminated encephalitis (ADEM), neurosarcoidosis, and primary CNS malignancy [5]. MRI spectroscopy can provide valuable information, and tissue diagnosis is often needed, especially in the absence of a previous history of MS and the absence of older demyelinating lesions on imaging.

Managing MS cases with an aggressive course or tumefactive lesions remains a challenge for clinicians. The effectiveness of first-line treatment with corticosteroids may be limited [6]. The efficacy of TPE for treating progressive MS was first reported in 1980, and since then, it has been studied for use in chronic progressive MS (CPMS) and acute relapses, with varying results. Multiple guidelines, including those from the American Academy of Neurology, the European Federation of Neurological Societies, and the American Society of Apheresis, recommend the use of plasmapheresis in neurologic conditions such as acute attacks of inflammatory demyelinating disease (IDD), primarily relapsing-remitting MS (RRMS), and neuromyelitis optica (NMO) [7,8].

The use of TPE and immunoadsorption has been used in patients with refractory MS and patients who don’t respond to steroids and reported a variable response rate that can reach up to 90% of cases [9]. Initiating TPE treatment early is linked to improved neurological outcomes and should therefore be considered as an early option when corticosteroids are not effective [6].

On biopsy, active demyelinating plaques can have four different histopathological patterns (patterns I-IV). Pattern II is characterized by excessive depositions of immunoglobulins with IgG predominance and complements, and the inflammatory lesions are believed to be driven mainly by lymphocytes and macrophages [10]. Pattern III shows mainly oligodendrocyte degenerative changes with inflammatory cells without immunoglobulin deposits, while Pattern I can have inflammatory cell depositions but also lacks significant immunoglobulin or complement deposits, and Pattern IV consists of oligodendrocyte death [10]. In one cohort study of 69 patients with biopsy-proven active MS who were treated with TPE, it showed that pattern II lesions had the best response to TPE [9]. The current American Society for Apheresis Guidelines recommend TPE in MS with acute, severe MS acute attacks or relapses that are not responsive to initial treatment with high-dose steroids [11]. It is worth mentioning that the severity of symptoms was not defined, but one can conclude that any neurological symptom that could adversely affect functional status could be considered severe.

Finally, patients with newly diagnosed TDLs who present with a clinically isolated syndrome have a higher risk of conversion to multiple sclerosis. One study found that >60% of patients who present with TDLs develop MS eventually [12]; thus, patients with isolated TDLs should be closely followed, both clinically and radiologically, and should be started on disease-modifying therapy once patients fulfil MS criteria.

## Conclusions

TDLs can offer a diagnostic challenge, especially in the absence of a history of multiple sclerosis. Given the size of the lesion and the possibility of a more sinister pathology, a biopsy and tissue diagnosis should be sought; additionally, the histopathological pattern of the demyelinating lesion could help in predicting the response to TPE. TPE offers more rapid removal of plasma-mediated antibodies and ultimately could provide a faster recovery. It is currently used as a second-line treatment for patients who fail to respond to initial steroid therapy. Additionally, patients with clinically isolated syndrome should be closely followed to determine the need for the initiation of disease-modifying therapy.
